# Ensemble feature selection with data-driven thresholding for Alzheimer's disease biomarker discovery

**DOI:** 10.1186/s12859-022-05132-9

**Published:** 2023-01-09

**Authors:** Annette Spooner, Gelareh Mohammadi, Perminder S. Sachdev, Henry Brodaty, Arcot Sowmya, for the Sydney Memory and Ageing Study and the Alzheimer’s Disease Neuroimaging Initiative

**Affiliations:** 1 School of Computer Science and Engineering, University of New South Wales, Sydney, Australia; 2 Centre for Healthy Brain Ageing (CHeBA), Discipline of Psychiatry & Mental Health, School of Clinical Medicine, University of New South Wales, Sydney, Australia

**Keywords:** Ensemble feature selection, Stability, Data-driven thresholding, Alzheimer's disease

## Abstract

**Background:**

Feature selection is often used to identify the important features in a dataset but can produce unstable results when applied to high-dimensional data. The stability of feature selection can be improved with the use of feature selection ensembles, which aggregate the results of multiple base feature selectors. However, a threshold must be applied to the final aggregated feature set to separate the relevant features from the redundant ones. A fixed threshold, which is typically used, offers no guarantee that the final set of selected features contains only relevant features. This work examines a selection of data-driven thresholds to automatically identify the relevant features in an ensemble feature selector and evaluates their predictive accuracy and stability. Ensemble feature selection with data-driven thresholding is applied to two real-world studies of Alzheimer's disease. Alzheimer's disease is a progressive neurodegenerative disease with no known cure, that begins at least 2–3 decades before overt symptoms appear, presenting an opportunity for researchers to identify early biomarkers that might identify patients at risk of developing Alzheimer's disease.

**Results:**

The ensemble feature selectors, combined with data-driven thresholds, produced more stable results, on the whole, than the equivalent individual feature selectors, showing an improvement in stability of up to 34%. The most successful data-driven thresholds were the robust rank aggregation threshold and the threshold algorithm threshold from the field of information retrieval. The features identified by applying these methods to datasets from Alzheimer's disease studies reflect current findings in the AD literature.

**Conclusions:**

Data-driven thresholds applied to ensemble feature selectors provide more stable, and therefore more reproducible, selections of features than individual feature selectors, without loss of performance. The use of a data-driven threshold eliminates the need to choose a fixed threshold a-priori and can select a more meaningful set of features. A reliable and compact set of features can produce more interpretable models by identifying the factors that are important in understanding a disease.

## Background

Healthcare datasets present many challenges to both machine learning and statistics alike. Their data are often heterogeneous, censored, high-dimensional and have missing information. In high-dimensional datasets, with a large number of features or variables and a small number of samples, typically only a small proportion of features may be relevant to the condition under investigation. Feature selection is generally used to reduce the dimension of the data, improve understanding of the problem and produce more interpretable models by identifying the factors that are important in understanding a disease [1]. A reliable model with fewer features can also lead to the development of more cost-effective procedures for identifying patients at risk of a disease.

However, applying feature selection to high-dimensional datasets often produces unstable results [2]. Stability, or reproducibility, of feature selection can be defined as the robustness of the selected features to perturbations in the data [3]. One of the key problems in modelling high-dimensional data, particularly where there are many redundant features, is the high variance of the models and feature selectors trained on this data. The same feature selection algorithm may select different subsets of features when run on different samples of the data while achieving a similar level of predictive accuracy [4]. If clinicians are to have confidence in machine learning models developed on healthcare datasets then the results of these models must be reproducible and therefore generalisable to new data.

Feature selection ensembles aggregate the results of multiple base feature selectors to improve stability and predictive accuracy [5]. One drawback of ensemble feature selectors, however, is that they do not provide a natural threshold to separate the relevant features from the irrelevant ones. A common method of setting this threshold is to choose a pre-determined fixed percentage of the number of features [6], but this offers no guarantee that the final set of selected features contains only relevant features. A data-driven threshold could overcome this problem and free the user from having to select and test different fixed thresholds [7].

Typically, simple univariate filters have been used as the base feature selectors in feature selection ensembles because they are computationally inexpensive, but they do not account for the interactions between features. More recently feature selection ensembles have been created from multivariate models [8, 9] and these models provide more opportunities for developing data-driven thresholds.

The focus of this work is to develop and test several data-driven thresholding methods in a novel context, that of ensemble feature selection using multivariate base feature selectors. The first method uses the 75% quartile of the feature importance scores as a threshold. The second method uses kernel density estimation (KDE) to cluster the feature importance scores and exclude the irrelevant features based on these clusters. The final method uses permutations of random probes [10], which are random features that have no association with the target variable, to identify the relevant features. While these methods have been used in other contexts, to the best of our knowledge they have not previously been applied to ensemble feature selection.

To demonstrate the applicability of these methods to clinical data, they are applied to data from two quite different real-world Alzheimer's disease (AD) studies—the Sydney Memory and Ageing Study (MAS) and the Alzheimer’s Disease Neuroimaging Initiative (ADNI). AD is a progressive neurodegenerative disease that results in declining cognitive function, such as memory, reasoning ability and executive function, and is ultimately fatal. Although the cause of this disease is not completely understood, the underlying pathological processes begin at least two to three decades before overt symptoms appear [11]. This presents an opportunity for researchers to determine early biomarkers that might help identify patients at risk of developing AD.

Ensemble feature selection has rarely been applied outside the context of classification. Data in healthcare, however, are often censored, meaning that the event of interest has not occurred during the study period, so the final outcome is unknown. Censored data are particularly common in AD studies as it is a slowly developing disease. The presence of censored data precludes the use of standard classification and regression techniques, but several machine learning algorithms have been adapted for survival analysis to handle censored data. It is these algorithms that we have chosen to investigate in this study, with the aim of expanding the use of ensemble feature selection to survival analysis. The methods developed here are applied to three real-world AD datasets to identify biomarkers for AD.

This work overcomes the limitations of previous studies on ensemble feature selection in several ways. First, previous studies have typically applied a fixed threshold to determine the final set of selected features. A fixed threshold does not adapt to different sized datasets and may include some irrelevant features or omit some important features. Our work overcomes this problem by developing data-driven thresholds that can automatically adapt to the size of the dataset under investigation and therefore eliminate the need to test different fixed thresholds. Second, ensemble feature selectors have often been constructed from simple univariate filters, which do not account for the interactions between features that are common in complex biological systems. Although more recent works have begun to use multivariate embedded feature selectors to overcome this problem, our work explores a number of feature selectors that have not been used in an ensemble of this type before, as well as adaptations of feature selectors for censored data. Finally, all previous studies referenced in this work have been applied to the task of classification only. Our work ensures that other tasks, including regression and survival analysis, can benefit from the superior performance and stability achieved by ensemble feature selection.

### Ensemble feature selection

Ensemble feature selectors apply a base feature selector to multiple subsamples of the training data, aggregate the results and apply a threshold to the resulting feature set. In the same way that bagging, boosting and stacking can improve the performance and reduce the variance of supervised learning methods, ensemble feature selection aims to improve the stability and predictive accuracy of the final subset of selected features [12]. The choice of the aggregation and thresholding methods are key elements that must be considered in the development of feature selection ensembles.

Ensemble feature selection was first proposed by Saeys et al. [6], who constructed homogeneous ensembles using simple filters applied to 40 bootstrap samples of the data and a simple linear sum of the feature rankings as aggregator. They compared the stability and performance of individual feature selection techniques with those of the ensembles and found that, in general, the ensemble techniques were more stable and had a similar predictive accuracy.

Other researchers have since conducted similar experiments, varying the feature selectors, the aggregators and the number of feature subsets in the ensemble [8, 9, 13–19]. The key characteristics of these studies and their contributions are summarised in Table 1.

**Table 1:** Key characteristics of ensemble feature selection studies referenced in this work

References	Ensemble structure	Feature selectors	Learners	Aggregation methods	Thresholds	Evaluation metrics	Data sets	Resampling strategy	Novelty
Saeys et al. [6]	Homogeneous, 40 bootstrap samples	Symetrical uncertainlty, Relief, RF, SVM RFE as	Linear SVM, RF, KNN	Linear sum of ranks	Fixed at 1% or 5% of # features	Accuracy, Spearman rank, Jaccard over 1% and 5%	3 Microarray, 3 mass spectrometry	Tenfold CV	1st to introduce Ensemble Feature Selection
Abeel et al. [13]	Homogeneous, 40 bootstrap samples	RFE—drop 20% at each iteration	Linear SVM	Linear sum of ranks, weighted linear sum of ranks	Fixed at 1% of # features	Kuncheva Index, AUC	4 Cancer diagnosis microarrays	Not given	Stability analysis, general experimental setup
Wald et al. [18]	Homogeneous, 50 bootstrap samples	10 filters: Chi-squared,IG, Relief, Mutual Information, Kolmogorov–Smirnov statistic, Deviance, Geometric Mean, AUCROC, AUCPROC and Signal-to-Noise	N/A	Mean rank, median rank, highest rank, lowest rank, stability selection, exponential weighting, enhanced borda, round robin, Robust Rank Aggregation	N/A	Consistency index	26 DNA microarray datasets	Not given	Compares similarity of methods, not performance
Brahim et al. [14]	Heterogeneous	CFS, Information Gain, ReliefF	KNN, decision tree, multilayer perceptron	Weighted mean rank, linear sum of ranks, proposed method	Fixed at 40 features	Precision, recall, F1-measure, AUC	3 Gene expression cancer datasets	Not given	New aggregation technique
Bolon-Canedo et al. [15]	Heterogeneous, aggregation before and after classification	5 filters: CFS, Consistency-based Filter, INTERACT, Information Gain and ReliefF	C4.5, naive Bayes, IB1 and SVM	Include only those subsets of features that outperform the classification accuracy in the training set	Fixed at 25 features	Mean test error, mean number of features required	1 Synthetic, 5 classic, 7 microarray	Tenfold CV	Heterogeneous ensembles, aggregation before and after classification
Brahim et al. [16]	30 bootstrap samples	3 filters: Relief, MRMR, t-test	KNN-1	Majority vote (ECA), WMA, CLA, Robust Rank Aggregation, frequency, CCA, RAA	Fixed at 1% of # features	Kuncheva Index, F-measure	7 Cancer microarray sets	Tenfold stratified CV	New aggregation technique -reliability assessment aggregation
Seijo-Pardo et al. [17]	Heterogeneous and homogeneous with 10 samples	3 filters and 2 embedded	SVM with Radial-Basis-Function	Min, median, mean, geometric mean, Stuart, Robust Rank Aggregation	Fisher discriminant ratio, log2(n), 10%, 25%, 50% of # features	Mean test error, mean training time	7 Datasets of varying types and sizes	Tenfold CV	Testing classification performance and runtime on a variety of datasets
Pes [9]	Homogeneous, 20, 50, 100 bootstrap samples, with 70, 80, 90% of original records included in training set	Chi-squared, Information Gain, Gain Ratio, OneR, ReliefF, SVM-AW, SVM-RFE	SVM, Random forest	Mean rank	Fixed at various values ranging from 0.2 to 40% of # features	AUC, consistecy index	18 Datasets of varying types and sizes	20 training/test sets	Investigates the patterns of stability and predictive performance for different subset sizes
Song et al. [8]	Homoogeneous, 20 bootstrap samples	ElasticNet, GBM, DNN	N/A	Mean rank, top-s, exponential top-s, these 3 weighted by OOB-AUC	Used an iterative golden-section search to estimate a minimal feature size	AUC, Kuncheva index, weighted and relative weighted consistency indices	Electronic medical records	Random samplling—10 times	Uses embedded feature selectors, rather than simple filters. Categorical and numeric data. Estimation of feature set size
Sechidis et al. [19]	Homogeneous, 50 samples, randomly removing 5% instances in each sample	Lasso, RF, GBM and filters MIM, mRMR, JMI, CMIM	3-nn	Weighted sliding-window average	Fixed at 25% of # features	Effective stability, nPOGR	13 Numerical UCI datasets of varying sizes	5% hold-out set, repeated 50 times	New stability measure and framework that account for feature correlations

Most existing studies of feature selection ensembles use only simple filters as base methods, as these are the most computationally efficient methods to apply to high-dimensional data. The filters chosen most frequently include chi-squared, information gain and ReliefF [9, 13, 18]. A notable exception [8] examines three sparse feature selectors, which return a subset of important features, including regularised regression, a tree-based gradient boosting machine and a deep neural network. Pes [9] and Sechidis et al. [19] also investigated a range of feature selection algorithms—univariate and multivariate, filters and embedded methods—in homogeneous ensembles in various application domains. But the use of multivariate feature selectors in ensemble feature selection is rare.

### Thresholding of feature selection ensembles

Much of the research on ensemble feature selection applies one or more fixed thresholds to the final feature selection in order to identify the most important features. In the case of gene rank aggregation, where the number of genes can run into the thousands or even tens of thousands, a threshold of 1% of the total number of features is common [6, 13, 16]. Various other values have been suggested, including log_2_(n) where n is the total number of features [17], 5% [6], 10% and 20% [9] of the total number of features.

As the number of relevant features is not known a-priori, a fixed threshold could include some irrelevant features or alternatively reject some relevant ones. Data-driven thresholding can potentially overcome this problem and also free the user from having to select and test different fixed thresholds for each model [7].

Very little work has been reported on developing an automatic or data-driven thresholding method for ensemble feature selectors and this is an open area of research. Some early related works used the "biggest gap" between consecutive aggregated values as a point of threshold [20]. While intuitively this method has merit, in practice it may result in a very small or a very large final feature subset and is not always reliable.

Seijo-Pardo et al. [20] experimented with the use of three different data complexity measures to set automatic thresholds. However, these measures are only applicable to classification and not to analysis of censored data.

Other researchers have investigated how to separate the important features from the redundant ones in individual feature selectors that return an importance score for each feature, such as random forests [10, 21–23]. Many of these methods use 'random probes' [21–23] to determine this boundary. A random probe is a random variable that has no association with the target variable and is typically created by randomly permuting the values of the existing features, thereby maintaining the same statistical distribution as the original features. Random probes are inserted as additional features into the data and the idea is that these features should be ranked last or at least as low as other irrelevant features. Features that are ranked below the probe can be discarded. The method of random probes is used in the popular Boruta feature selector [23]. Huynh-Thu et al. [10] were the first to suggest examining random probes in the context of stability analysis.

One-dimensional clustering of the feature importance scores is another method that may be useful in determining a threshold. The popular K-Means clustering method [24] has several limitations that make it unsuitable for this application, however. It requires the specification of the number of clusters a-priori, cannot detect non-spherical clusters and does not account for cluster density [25]. MAP-DP [25] is an alternative to K-means and was developed to overcome the limitations of K-means. It can handle clusters of different shapes and determines the number of clusters from the data, but it requires many other parameters to be specified and so needs an in-depth knowledge of the algorithm.

Rodriguez and Liao [39] proposed a method of non-parametric clustering using kernel density estimation (KDE), that is able to find the correct number of clusters and detect non-spherical clusters. The cluster centres are defined as the local maxima in the density of data points. Once the centres have been identified, each point is assigned to the same cluster as its nearest neighbour of higher density. KDE overcomes the limitations of K-means clustering [24] and MAP-DP [25].

### Aggregation of feature selection ensembles

The problem of combining several ranked lists into a single final ranked list has been studied in fields as diverse as information retrieval, voting theory and bioinformatics, and various techniques have been proposed [18]. In statistics, this is known as the consensus ranking problem—"given m rankings of n objects, which ranking best represents the consensus opinion?" [26]. The *m* rankings may contain ties, be incomplete, and may be weighted.

Simple mathematical combinations, such as the mean, median or sum of the feature ranks or weights, are effective aggregation techniques and widely used [6, 13, 14, 16]. A count of the number of times each feature is selected by the base methods is also a commonly used method [16, 27]. Intuitively, if a feature is consistently given a high ranking in different data samples or by different methods, then it is likely to be important.

Wald et al. [18] carried out an extensive comparison of nine different rank aggregation techniques across twenty-six bioinformatics datasets and noted certain similarities between them. For example the Borda Count [28], a method often used in voting theory, is mathematically equivalent to the arithmetic mean and will rank features in the same order.

In some cases aggregation and thresholding are combined. Kolde et al. [29] proposed an algorithm called Robust Rank Aggregation (RRA) for prioritised lists of genes, that assigns a significance score for each gene. It determines the probability that a randomly generated rank list would have scored that feature more highly. The lower this probability, the more important the feature is. This method not only ranks the features but provides a statistically relevant threshold as well.

RRA can be expressed as follows. If r is an ordered vector of ranks and $\hat{r}$ is the rank vector generated by the null model (i.e. sampled from the uniform distribution), then under the null model, the probability that $\hat{r}\left( k \right) \le x$ can be expressed as a binomial probability:

1 $$\beta_{k,n} \left( x \right): = \mathop \sum \limits_{l = k}^{n} \left( {\begin{array}{*{20}c} n \\ l \\ \end{array} } \right)x^{l} \left( {1 - x} \right)^{n - l}$$

Then the final score for the rank vector r is defined as the minimum of p-values:

2 $$\rho \left( r \right) = \mathop {\min }\limits_{k = 1, \ldots ,n} \beta_{k,n} \left( r \right)$$

In the field of information retrieval, the number of items being ranked is usually very large and so it is not feasible to access every item in the database to calculate an aggregated score. More efficient techniques are needed for tasks such as the ranking of web search results or legal documents. The threshold algorithm (TA) is a simple yet elegant algorithm that allows early stopping and yields the top *k* features, where *k* must be chosen in advance [30]. Elements are accessed sequentially from the ranked lists i.e. the first element of each list is examined first, then the second element of each list and so on. At each sequential access a threshold equal to the sum of the scores in that access is set. For any items seen in the current access, random accesses are made to sample a set of scores for that element and an aggregate is calculated. A list of the highest scoring *k* items seen so far is maintained and when all items in that set are greater than or equal to the threshold, the algorithm terminates. Therefore, it performs both aggregation and thresholding.

Another algorithm from the domain of information retrieval that can perform both aggregation and thresholding is the MedRank algorithm [31]. The MedRank algorithm also accesses the rankings sequentially. When an element has appeared in more than half of the ranked lists, it is output to the aggregated ranking. The algorithm can terminate early if only the top *k* rankings are required.

This work proposes three different data-driven thresholding techniques for ensemble feature selection, adapted from other areas of research and applied in a novel context. These techniques are tested with two different real-world AD datasets and compared to a selection of fixed thresholds.

### Stability measures

Somol and Novovičová [32] studied measures of feature selection stability and noted several desirable properties. The measure should be bounded by 0 and 1 where a value of 1 should imply a high level of stability, whereas a value of 0 should imply a low level of stability. The measure should also be capable of evaluating the stability of feature sets of varying sizes.

Kuncheva's *stability index* has been used by several authors investigating the stability of feature selection subsets [8, 13, 16]. The index for two subsets S_1_, S_2_ is defined as:

3 $$I_{c} \left( {S_{1} , S_{2} } \right) = \frac{{rn - k^{2} }}{{k\left( {n - k} \right)}}$$

where n is the total number of features, k is the size of the two sets and r is the size of the intersection of the two sets. This index can only be applied to subsets of identical size.

Lustgarten's *adjusted stability measure* [33] is an improvement on Kuncheva’s measure in that it handles subsets of varying sizes, but does not fulfil the other desired properties. Lustgarten’s measure can be defined as

4 $$S_{A} \left( {S_{i} , S_{j} } \right) = \frac{{r - \frac{ki kj}{n}}}{{\min \left( {ki, kj} \right) - {\text{max}}\left( {0, ki + kj - n} \right)}}$$

Somol and Novovičová's [32] *relative weighted consistency* of a set of feature subsets is an ideal measure as it meets the desired properties and does not overemphasise low-frequency features. The relative weighted consistency $CW_{rel} \left( {S, Y} \right)$ of system S characterised by N, n and for given Y is defined as:

5 $$CW_{rel} \left( {S,Y} \right) = \frac{{\left| Y \right|\left( {N - D + \mathop \sum \nolimits_{f \in Y} F_{f} \left( {F_{f} - 1} \right)} \right) - N^{2} + D^{2} }}{{\left| Y \right|\left( {H^{2} + n\left( {N - H} \right) - D} \right) - N^{2} + D^{2} }}$$

where $\left| Y \right|$ is the total number of features, F_f_ is the number of occurrences (frequency) of feature $f \in Y$ in system S, N is the total number of occurrences of any feature in system S, n is the number of feature sets and $D = n mod \left| Y \right|, H = N mod n.$

## Methods

### Study cohort

Experiments in this work were conducted on data from two real-world AD datasets—the Sydney Memory and Ageing Study [34] and the Alzheimer's Disease Neuroimaging Initiative [35]. The characteristics of both studies are summarised in Table 2 and a brief description of each is given in the sections titled "Sydney memory and ageing study (MAS)" and "Alzheimer's disease neuroimaging initiative (ADNI)". Full details can be found in the references provided.

**Table 2:** Study characteristics

	MAS	ADNI-1
Study design	Population based cohort study	Multisite longitudinal study
Sample size (n)	873	819
Number of features (p)	140	216
Censoring rate	93%	47%
Intervals between waves	2 years	After 3, 6, 12, 18, 24, 36 and 48 months
Age at baseline	70–90 years	55 – 90 years
Number of cases of AD	64	437

The two datasets are quite different in terms of their study cohorts, data collected and depth of investigation and as such their features are not comparable. Instead, the aim in applying the methods developed here to these two datasets is to demonstrate their applicability to clinical data.

#### Sydney memory and ageing study (MAS)

The Sydney Memory and Ageing Study (MAS) is a population-based cohort study aimed at examining the characteristics and prevalence of mild cognitive impairment and dementia. Full details of the study can be found [34]. The MAS data set contains a diverse collection of data including demographics, genetics, cognitive data, medical history, family history, medical examination, blood test results, psychological scores and functional data. Data that were used in forming a diagnosis of AD have not been used in the models developed here to predict AD.

The experiments reported here used only the baseline data, collected in the first wave of MAS. Participants from a non-English-speaking background were excluded, leaving 873 participants from the original 1037. The event of interest in the survival analysis was a diagnosis of possible or probable Alzheimer's disease, over a period of 6 years, from wave 1 to wave 4 of the study. During this period 64 people developed Alzheimer’s disease, indicating a censoring rate of 93%.

The Human Research Ethics Committees of the University of New South Wales and the South Eastern Sydney and Illawarra Area Health Service granted ethics approval for the MAS study and informed written consent was given by all participants and informants. The MAS study and this work were carried out in accordance with the MAS Governance guidelines, which are based on relevant University of New South Wales and National Health and Medical Research Council research and ethics policies.

#### Alzheimer's disease neuroimaging initiative (ADNI)

The ADNI was launched in 2003 as a public–private partnership, led by Principal Investigator Michael W. Weiner, MD. The primary goal of ADNI has been to test whether serial magnetic resonance imaging (MRI), positron emission tomography (PET), other biological markers, and clinical and neuropsychological assessment can be combined to measure the progression of mild cognitive impairment (MCI) and early Alzheimer’s disease (AD).

ADNI participants were aged 55–90 years at enrolment and were recruited from 57 sites in the United States and Canada. The ADNI data set contains data from a clinical evaluation, neuropsychological tests, genetic testing, lumbar puncture, and MRI and PET scans. Subjects who participated in ADNI phase 1 were selected for this study. The event of interest in the survival analysis was a diagnosis of probable AD, over the period of the ADNI 1 study. A total of 200 participants with early AD were enrolled at the start of the study and a further 237 participants developed AD during the course of the study. Data that were used in forming a diagnosis of AD, have not been used in the models developed here to predict AD.

### Experimental framework

To prepare the data for the feature selection algorithms, missing data were imputed using the method of multiple imputation by chained equations in the R package *mice* [36]. Imputation was performed within the cross-validation loop.

Continuous features were normalised, by subtracting the mean and dividing by the standard deviation, and multiple values for the same measurement, e.g. blood pressure, were averaged. Levels of categorical features containing only a small number of samples were combined where possible. Further details of pre-processing steps can be found [37].

The R [38] package *mlr* (Machine Learning in R) [39] was used as a basis to carry out the experiments, while customised code was written to construct the ensembles. All of the ensembles were constructed within a fivefold cross-validation framework, repeated 5 times. Random probes were generated for each subsample of the data. Experiments were performed on the computational cluster Katana, supported by Research Technology Services at UNSW Sydney [40].

### Base feature selectors

The ensemble feature selectors were constructed from six different base feature selectors, each capable of selecting features from high-dimensional, heterogeneous, censored data. Four sparse methods, which return a subset of important features, and two filter methods, returning a score for each feature, were chosen. The four sparse methods were penalised regression for the Cox model (specifically the LASSO [41] and the ELASTIC-NET [42]), the Cox model with gradient boosting (GLMBOOST [43]) and the Cox model with likelihood-based boosting (COXBOOST [44]). The two filter methods were the maximally selected rank statistics random forest (RANGER [45]) and a univariate Cox filter (UNI). Each represents a different style of feature selection algorithm. The Cox filter was the only univariate method—the others are all multivariate feature selectors.

The absolute values of the coefficients of the features were used as feature importance scores for the sparse models—the LASSO, ELASTIC-NET, GLMBOOST and COXBOOST. These coefficients are meaningful importance scores because the data were normalised within the cross-validation loop prior to modelling. The other two models provide a feature importance score for each feature. For the RANGER, this was calculated using the method of permutation importance and for the univariate filter, the feature importance score was the value of the C-Index returned by a Cox Proportional Hazards model applied to each feature individually.

Further information about the functioning of these methods and the R packages used to implement them can be found [37].

Each of these feature selectors was first tested in its individual form. Within a framework of 5 repeats of 5-fold cross validation, the feature selector was applied to the training data to select relevant features. Several fixed thresholds (10%, 25%, 33% of the total number of features) were applied to the results of the filter methods, but as the sparse methods already select a subset of features, no further thresholding was applied to their results. A Ridge survival analysis model was trained and tested on the reduced dataset and the performance and stability of the individual models were compared to those of the ensemble models. The Ridge was chosen because of its superior performance in previous experiments [37].

### Ensemble construction

**Fig. 1:**
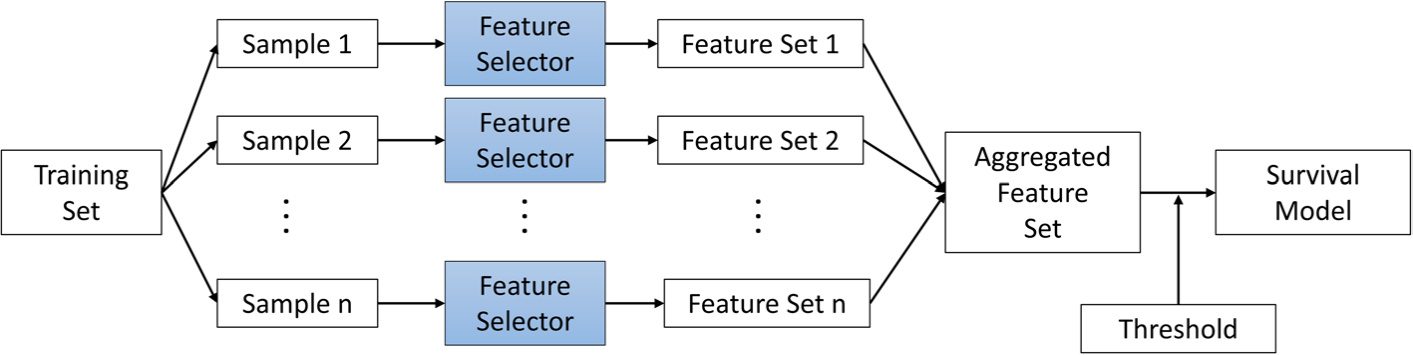
A homogeneous feature selection ensemble. Sample1, Sample 2 … Sample n are randomly sampled subsets of the training data. The same feature selector is applied separately to each sample, generating n sets of selected features. An aggregator is applied to combine these feature sets into a single set, a threshold is applied and the resulting feature set is used as input to a survival model to assess its accuracy

Homogeneous feature selection ensembles were constructed by applying the same feature selector to 50 bootstrapped samples of the training data, producing 50 subsets of features, as shown in Fig. 1. An aggregator was used to combine these feature subsets into a single set and the resulting feature set was used as input to a machine learning model, in this case a survival analysis model, to assess its accuracy.

Five different aggregators were used to combine the feature subsets into a final feature set. Three of these aggregators also perform thresholding:

1. *Mean rank (MR)*: The features in each subset were ranked according to their feature importance scores or weights and the mean of these ranks across all subsets was taken as the final aggregated score. The features were again ranked by this aggregated score.
2. *Mean weight (MW)*: The weights or feature importance scores were averaged across all feature subsets and the features were ranked by the aggregated score.
3. *Robust Rank Aggregation (RRA)* [29]: This method calculates a p-value, a statistically significant threshold, for each feature. A range of different p-values was tested and in each case only features with a p-value less than that being tested were selected. The abbreviations RRA05, RRA10, RRA15, RRA20 and RRA25 refer to the RRA method tested with p-values of 0.05, 0.1, 0.15, 0.2, 0.25 respectively. This method performs both aggregation and thresholding, so no other thresholding techniques were applied.
4. *Threshold Algorithm (TA)*: In each fold of the training data, the number of items to be returned, *k*, was set as the mean length of the 50 feature subsets generated by the ensemble. Elements were accessed sequentially from the ranked lists. At each sequential access a threshold equal to the sum of the scores in that access was set. A list of the highest scoring *k* items seen so far was maintained. When all items in that set were greater than or equal to the threshold, the algorithm terminated. This algorithm performs both aggregation and thresholding, so no other thresholding techniques were applied
5. *Medrank Algorithm (MA)*: In each fold of the training data, **t**he number of features to be returned, *k*, was set as the mean length of the 50 feature subsets generated by the ensemble. Features were accessed sequentially from the ranked lists. When a feature appeared in more than 20% of the ranked lists, it was output to the aggregated list. (Here we used 20% rather than 50% as in the original algorithm because the signal is weak and very few features appear in more than half the lists). If the length of the aggregated list reached *k*, the algorithm terminated early.

A mixture of fixed and data driven thresholds were tested for comparison. Three different fixed thresholds were chosen—10%, 25% and 33% of the total number of features, applied after aggregation.

Three different data-driven thresholds were also tested. The first method used the 75% quartile of the feature importance scores as a threshold. In preliminary testing the 25% and 50% quartiles were also examined, but the 75% quartile provided consistently better results, and so only that method has been included here.

**Fig. 2:**
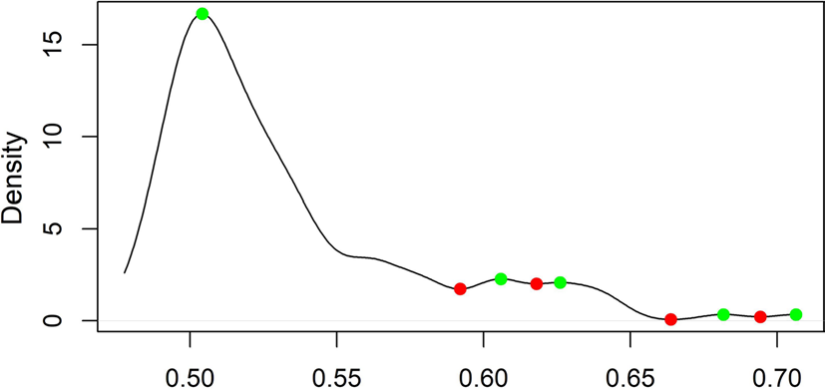
Example plot of kernel density estimate for one-dimensional clustering. The green dots show the local maxima, which are the cluster centres. The red dots show the local minima, which are the cluster boundaries. The maximum of the local maxima is the cluster centre for the irrelevant features

The second method used kernel density estimation (KDE) to cluster the 1-dimensional importance scores, and this is the first time KDE has been used as a threshold in the context of ensemble feature selection. The cluster centres are defined as the local maxima in the density of data points. Each point is assigned to the same cluster as its nearest neighbour of higher density. In the case of 1-dimensional data, KDE can be plotted, as shown in Fig. 2. The green dots show the local maxima or cluster centres. The red dots show the local minima, which are the boundaries between clusters. A key assumption of the method proposed here is that the majority of features are irrelevant, which is often the case in high-dimensional data. Then the maximum peak in the kernel density plot will be the cluster centre for the irrelevant features. Therefore, any features with an importance score higher than the upper boundary of that cluster (i.e. higher than the next local minimum), are the relevant features.

KDE determines the number of clusters from the data but still requires a bandwidth (also called a smoothing parameter) to be selected. Here the well-known rule of thumb, Silverman's rule, was used to select the bandwidth [46]. If the selected bandwidth was too large and produced only a single local maximum, and no local minima, a smaller value was generated by multiplying the original value by a factor of 0.75, and the density estimation was repeated. If a valid value was not found, no thresholding was performed.

The final method of data-driven thresholding tested used random probes to determine the boundary between the relevant and irrelevant features [10, 21–23]. A random probe is a random variable that has no association with the target variable and is typically created by randomly permuting the values of the existing features, thereby maintaining the same statistical distribution but breaking the correlation with the target variable. The values of the probes were randomly permuted on each of the 50 iterations. Any feature that was ranked below the rank of the highest random probe rank was considered irrelevant. This is the first time that random probes have been used in the context of ensemble feature selection, although a similar idea was suggested by Huynh-Thu et al. [10].

Random probes were originally developed for use with continuous numeric data and feature selectors that return a score for each feature, so some adjustment was necessary to use them with heterogeneous data and sparse feature selectors. First, it is possible that a sparse feature selector may not select any of the random probes, meaning that none are given a feature importance score, and therefore a comparison cannot be made between the importance of the probes and the importance of the features. In this case all of the features selected by the sparse feature selector were considered relevant. Second, randomly permuting Boolean features (with values limited to TRUE or FALSE) and categorical features (with values limited to a small set of possible values) can leave some values in the same position, and so some of the random probes can achieve quite high importance scores, potentially eliminating truly important features. The random probes generated from Boolean features were excluded from the dataset for this reason. However, the random probes generated from categorical features were retained as the larger number of possible values ensured a more random permutation.

### Performance metrics

The prediction accuracy of the feature selection ensembles was assessed by the value of the Concordance Index (C-Index) achieved by a RIDGE survival model trained on the features selected by the ensemble. The Ridge model was chosen for its superior performance and stability in prior experiments [37]. The C-Index measures the proportion of pairs where the observation with the higher actual survival time has the higher probability of survival as predicted by the model [47]. The performance score for the ensemble was the mean of the performance scores over five repeats of 5-fold cross validation.

Each ensemble feature selector or individual method generated 25 final feature subsets from the 5 repeats of 5-fold cross validation. The stability of each ensemble was measured by applying Somol and Novovičová's *relative weighted consistency* [32] to these 25 feature subsets. This metric was chosen as it is capable of evaluating feature selectors that yield subsets of varying size.

## Results

The aim of this work is to develop and test a variety of data-driven thresholds for use with homogeneous feature selection ensembles, so as to free the user from having to select a fixed threshold. Three methods of data-driven thresholding were applied in a novel context and evaluated—the 75% quartile of the feature importance scores, KDE and the best random probe score. Three existing methods of thresholding, that combine thresholding with aggregation, were also tested—the RRA, and the Medrank (MA) and Threshold (TA) algorithms from the field of information retrieval. Three fixed thresholds were included for comparison. The thresholding methods were applied to homogeneous feature selection ensembles constructed from six different base feature selectors, capable of handling high-dimensional, right-censored data, and the stability and predictive accuracy of the ensembles were compared to their equivalent individual form. The six base feature selectors were penalised regression for the Cox model (specifically the LASSO [41] and the ELASTIC-NET [42]), the Cox model with gradient boosting (GLMBOOST [43]), the Cox model with likelihood-based boosting (COXBOOST [44]), the maximally selected rank statistics random forest (RANGER [45]) and a univariate Cox filter (UNI). The features selected by these models were also examined as possible biomarkers for Alzheimer's disease.

**Fig. 3:**
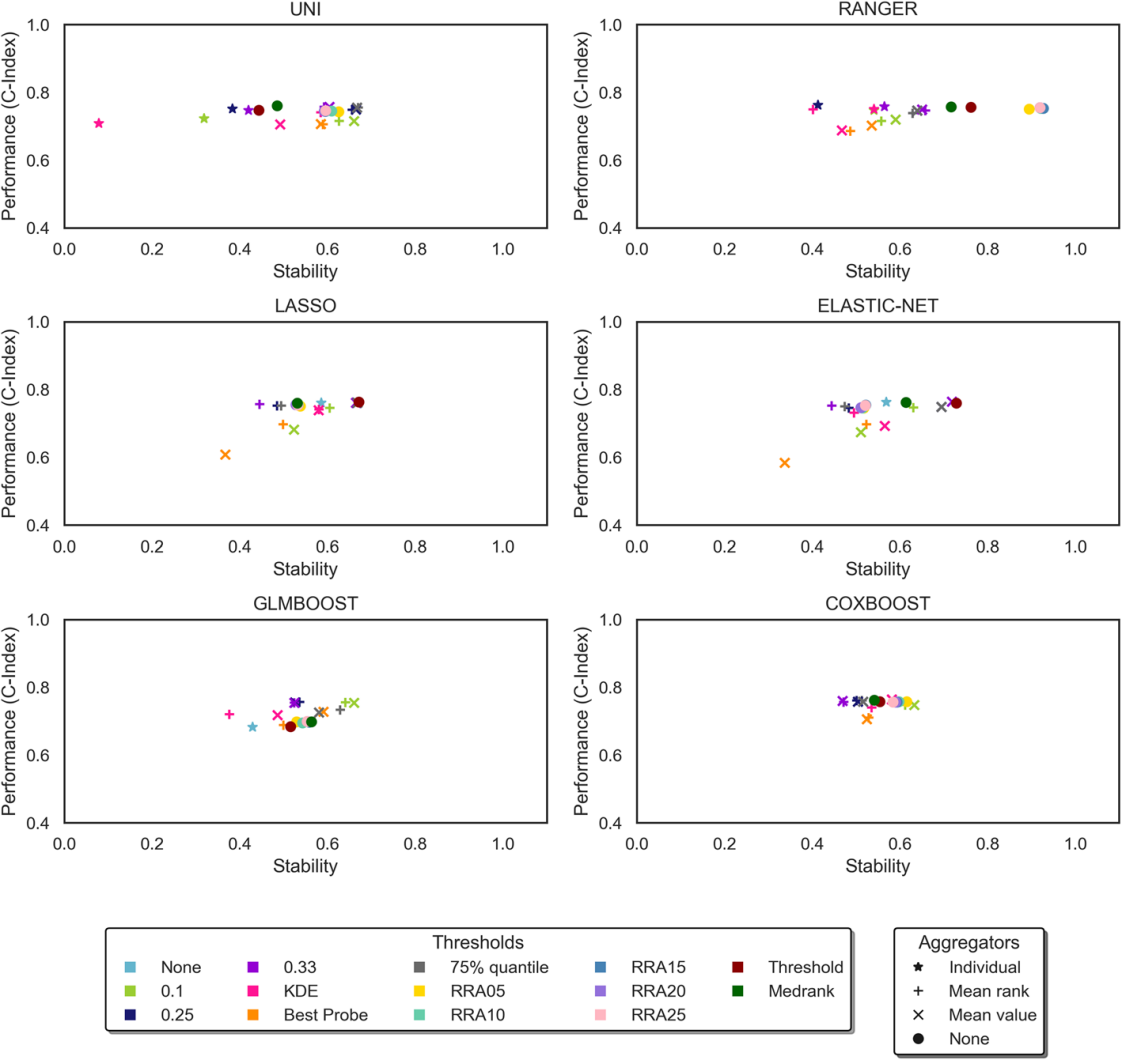
Experimental results from the MAS dataset. Each plot shows performance vs stability of one feature selector. The different shapes represent different aggregators, with a star shape representing the individual form, where the model is run only once and there is no aggregation of results. The different colours represent the different thresholds applied to the models. The abbreviations RRA05, RRA10, RRA15, RRA20 and RRA25 refer to the RRA method tested with p-values of 0.05, 0.1, 0.15, 0.2, 0.25 respectively

**Fig. 4:**
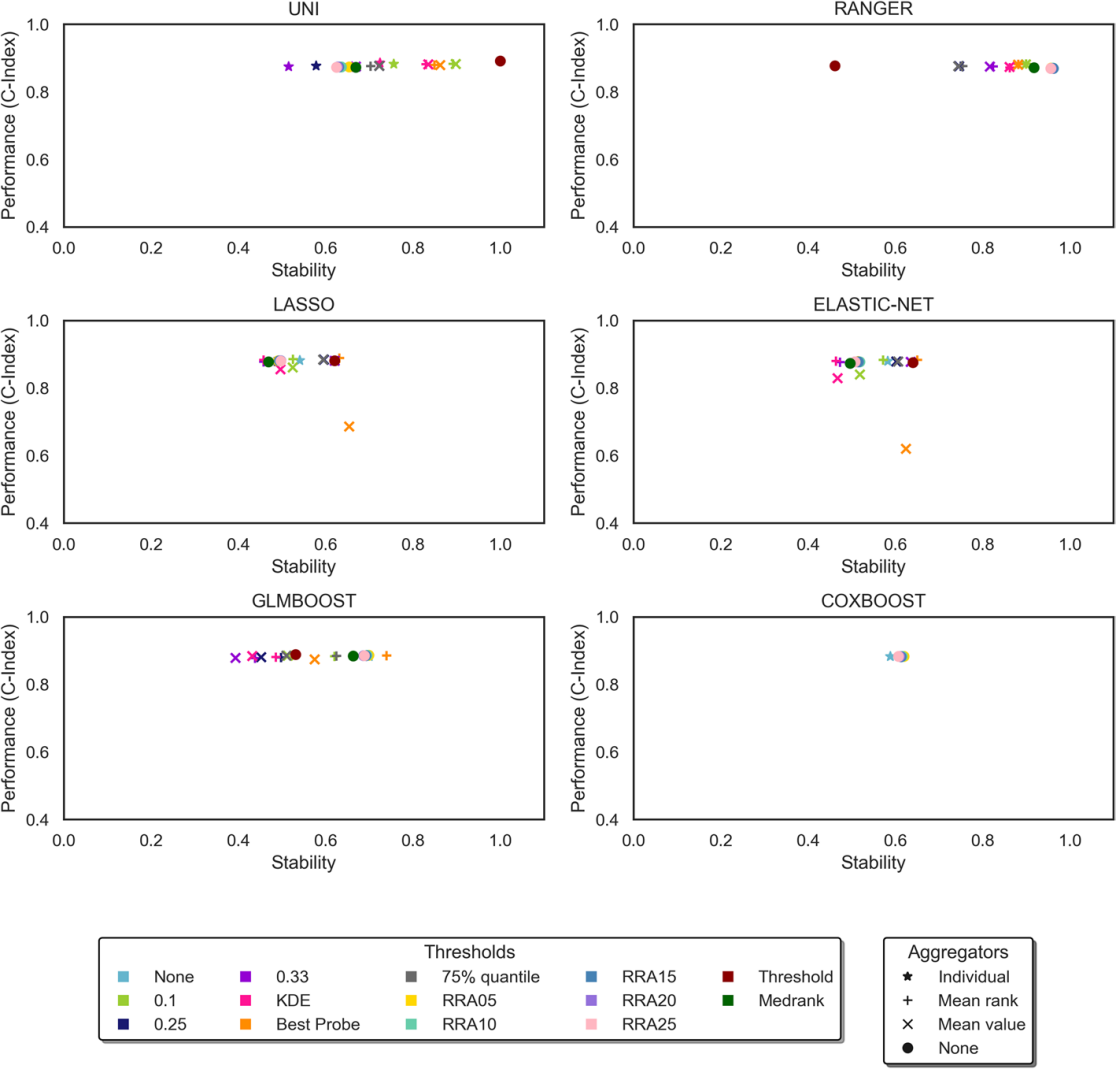
Experimental results from the ADNI dataset. Each plot shows performance vs stability of one feature selector. The different shapes represent different aggregators, with a star shape representing the individual form, where the model is run only once and there is no aggregation of results. The different colours represent the different thresholds applied to the models. The abbreviations RRA05, RRA10, RRA15, RRA20 and RRA25 refer to the RRA method tested with p-values of 0.05, 0.1, 0.15, 0.2, 0.25 respectively

Following the method of Song et al. [8], the predictive accuracy of the models was plotted against their stability in the graphs in Fig. 3 for the MAS dataset and Fig. 4 for the ADNI dataset. Values further to the right of the graph indicate more stable models and values higher on the graph indicate models with a higher predictive accuracy.

Note that the sparse feature selectors are designed to select a subset of the most useful features, therefore in their individual form no further thresholding is applied and there is only a single result for the individual form of the model (represented by a pale blue star). However, in the case of the filter methods, which return a score for each feature, a threshold must always be applied, even in the individual form, to select the most useful features. Therefore, the filter methods have multiple results for the individual form of the model, one for each fixed threshold and for KDE.

Song et al. [8] compared the overall performance of the models using the Euclidean distance from the origin in the plots of predictive accuracy vs stability. Using this method, the best ensemble model overall for the MAS dataset was the RANGER model with the RRA threshold using a p-value of 0.15, and the best ensemble model for the ADNI dataset was the UNI model with the TA threshold. Both of these models use a data-driven threshold.

The graphs in Figs. 3 and 4 show that most of the ensemble models are more stable than the individual form of the same model, with a value further to the right on the graph. In the MAS dataset, the greatest improvement in the Euclidean distance from the origin is once again seen in the RANGER model with the RRA threshold using a p-value of 0.15. In this case the ensemble shows an increase of 0.29 or 34% over the least stable individual model. In the ADNI dataset, the UNI model with the TA threshold shows the greatest improvement, with an increase of 0.32 or 32% over the least stable individual model. These improvements are mainly due to improved stability, which can be observed in the graphs in Figs. 3 and 4.

**Fig. 5:**
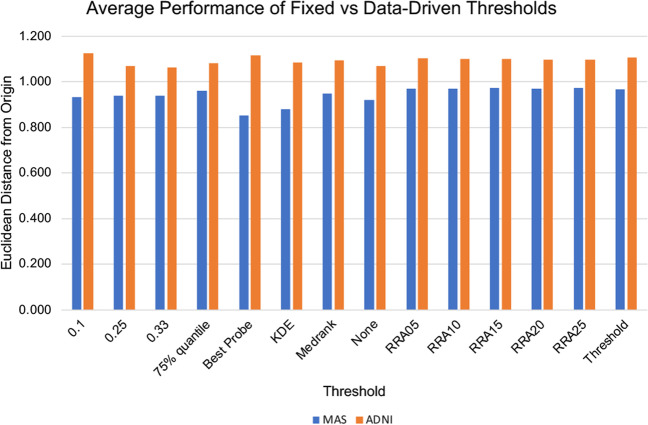
Average Euclidean distance from the origin for each threshold for the MAS and ADNI datasets

The best threshold overall was determined by taking the average Euclidean distance from the origin for all models using each threshold. These results can be seen in the graph in Fig. 5 and in Table 3 where the thresholds are listed in order of performance. In the MAS dataset the top four performing thresholds (including all variations of RRA as one threshold) are data-driven thresholds, and these outperform the three fixed thresholds, while in the ADNI dataset the 10% fixed threshold is the top performing threshold, but the other two fixed thresholds are the worst performing. The RRA aggregator showed similar performance across the range of p-values tested.

**Table 3:** Average Euclidean distance from the origin for each threshold in the ADNI and MAS datasets, ordered from best to worst in each dataset, to show the relative performance of the methods

MAS		ADNI	
RRA15	0.973	0.1	1.125
RRA25	0.972	Best Probe	1.116
RRA10	0.971	Threshold	1.106
RRA05	0.971	RRA05	1.103
RRA20	0.970	RRA10	1.102
Threshold	0.968	RRA15	1.100
75% quantile	0.960	RRA20	1.098
Medrank	0.947	RRA25	1.097
0.33	0.939	Medrank	1.094
0.25	0.939	KDE	1.086
0.1	0.933	75% quantile	1.082
None	0.920	None	1.069
KDE	0.881	0.25	1.069
Best Probe	0.852	0.33	1.064

The fixed thresholds show varying performance across the two datasets, showing that a fixed threshold must be tailored to the data at hand and carefully selected. In the MAS dataset, the performance of the three fixed thresholds is in the middle of the range, while in the ADNI dataset, which has a different number of features and samples, the fixed thresholds exhibit both the best and worst performance. It is clear that a fixed threshold must be carefully chosen to suit the dataset under investigation.

### Interpreting the features selected

The features selected by the ensemble feature selectors may be indicative biomarkers for AD. However, because of the stochastic nature of machine learning, even with improved stability each combination of feature selector, aggregator and threshold can still return a slightly different set of features. So, a decision must be made as to which is the optimal set of selected features.

Here the optimal features have been identified as those selected in at least half of the top 10 performing models. In the case of the MAS dataset, this metric was restricted further to features selected in at least 80% of the top performing models, as a large number of features were selected. Features that are selected consistently by models that perform well are likely to be important features. Using this method, the features selected using data-driven thresholding from the MAS dataset are described in Table 4 and from the ADNI dataset in Table 5. The AD literature supports the findings from both datasets and references are given in Tables 4 and 5 to demonstrate this.

**Table 4:** Features selected from the MAS dataset by the best models using data-driven thresholding

MAS feature descriptions	AD biomarker evidence
Participant age at time of testing	The most important predictor of dementia [48]
Status of the epsilon4 allele of the APOE gene	Increases the risk of late-onset AD [11]
Waist to hip ratio	Obesity and cardiovascular risk factors have been linked to dementia [49]
Framingham cardio-vascular risk score	
General Practitioner Assessment of Cognition score (GPCOG)	Designed to identify dementia [50]
Mini Mental State Exam score (MMSE)	Designed to identify dementia [51]
Informant Questionnaire on Cognitive Decline in the Elderly (IQCODE)	Designed to identify dementia [52]
Informant subjective cognitive complaints – total score	Increasingly being recognised as predictors of progression to mild cognitive impairment and dementia [53]
Participant subjective cognitive complaints – total score	
Composite variable encoding the number of major and minor at fault motor vehicle accidents in the past 18 months	Evidence exists that atypical changes in driving behaviours may be early signs of mild cognitive impairment (MCI) and dementia [54] [55]
Normal or abnormal posture	Abnormal posture or gait can be related to increasing frailty, which is associated with dementia [56]
Normal or abnormal gait	
Urinary tract infection	Urinary tract infections are known to exacerbate dementia symptoms
Arthritis	A recent review highlighted the link between rheumatoid arthritis and dementia [57] although other researchers have reported a negative link with AD rather than dementia generally [58]
Urate	Studies have found a link between low serum uric acid levels and AD [59]
Uric acid

**Table 5:** Features selected from the ADNI dataset by the best models using data-driven thresholding

ADNI feature descriptions	AD biomarker evidence
Mini Mental State Exam score (MMSE)	Designed to identify dementia [51]
Instrumental activities of daily living (IADL) score	Often used to determine the cognitive function of an individual [60]
Status of the epsilon 4 allele of the APOE gene	Increases the risk of late-onset AD [11]
42-amino-acid-long beta amyloid peptide (abeta42) level in CSF	Thought to be a biomarker for AD [61]
Plasma neurofilament light level	Increased concentrations of CSF and plasma levels of the neurofilament light chain have been identified as potential biomarkers of AD [62] [63]
CSF neurofilament light level	
CSF tau level	The presence of tau and phosphorylated tau in CSF accurately detects Alzheimer's disease pathology [64]
CSF phosphorylated tau level	
Neuropsychiatric Inventory – total score	Can be used to detect behavioural changes in AD [65]
CSF neurogranin concentration	Patients with AD show an increase in CSF neurogranin concentration and this is not seen in other neuro- degenerative diseases [66]
Geriatric depression scale – total score	Depression has been linked to AD and cognitive decline [67]
Percentage of neutrophils	People with AD have a higher blood neutrophil–lymphocyte ratio (NLR), a marker of inflammation, than healthy controls [68]

It should be noted that there are significant differences between the ADNI and MAS datasets. MAS is a population-based study which recruited non-demented individuals randomly from the community, while ADNI recruited people with a diagnosis of AD or cognitively healthy individuals as volunteers. There are also differences in the data collected by these studies. ADNI had a strong focus on neuroimaging (structural MRI and PET) and biomarkers from cerebrospinal fluid (CSF) and plasma. In contrast, MRI imaging was performed on only a subset of MAS patients, PET imaging was not performed, no CSF was collected, and fewer blood tests were performed in the baseline waves of MAS. MAS had a stronger focus on medical history and examination, psychological and neuro-psychological assessment, and self-administered lifestyle questionnaires. Therefore, the features selected in the two datasets differ to some extent.

## Discussion

The development of a data-driven threshold that can be applied to ensemble feature selectors for both censored and uncensored data is a distinct advantage. A fixed threshold that is appropriate for one data set may be quite inappropriate for another, especially when those datasets vary greatly in terms of the number and type of features they contain. The successful use of a fixed threshold involves testing of a wide range of fixed thresholds during the development of each model and can be quite time consuming.

The data-driven thresholds tested here performed well, being amongst the top performing thresholds in both datasets. The RRA threshold has the added advantage that it provides a *p*-value—a statistically relevant threshold, and so its use is highly recommended, particularly with clinical data. The RRA threshold was tested with a range of different *p*-values, but there was little to differentiate the performance of the different p-values in either dataset.

The disadvantage of the KDE threshold is that it requires that the data contain a large number of irrelevant features to be successful. The KDE threshold performs better in the ADNI dataset than in the MAS dataset. This is because the ADNI dataset contains more features and so it is likely that it also has more irrelevant features.

The Best Probe threshold with the mean value aggregator did not perform well with the LASSO or ELASTICNET in either dataset. On investigation it was found that a random probe was often one of the most important features selected by the LASSO, thereby eliminating other truly important features. This could be the result of correlations in the data as the Lasso is known to select one feature at random from a group of correlated features. Further investigation is warranted to clarify this.

The Medrank and Threshold algorithms are primarily aggregators, and both output a fixed number of features, set in this case as the mean length of the ranked lists of features. Therefore, varying this number could affect their performance. Despite this, both perform well in these experiments, demonstrating the importance of an effective aggregation strategy.

As well as investigating different thresholds, a number of different feature selectors were examined in this work, including simple filters, penalised regression, random forests and boosted models. Although most previous works on ensemble feature selection have used only filters, it is clear from these results that multivariate feature selectors can also benefit by being used in an ensemble.

The ADNI dataset exhibited better performance and stability than the MAS dataset, which was to be expected. ADNI is an observational cohort study, where the number of patients with AD was carefully controlled at the start. Not only are there more cases of AD in ADNI, giving it a much lower censoring rate than MAS, but participants with AD at baseline were accepted into the study. In contrast, MAS is a population-based cohort study, where participants were excluded if they had AD at baseline, and so the number of AD cases cannot be controlled and there are fewer cases of AD than in ADNI.

In future it would be of interest to train the models on one AD dataset and apply them to another AD dataset with a comparable cohort and set of features, but the aim here was to demonstrate the applicability of the methods to different datasets, albeit in the same area. The methods were applied to a third dataset, which had few events and fewer samples than the other datasets. The results were mixed and have not been included here but they demonstrate the need for an adequate amount of data for these methods to succeed.

Future work could investigate the use of the Knockoffs method for variable selection [69], rather than random probes. Like random probes, knockoffs are random variables that have no association with the target variable. Whereas random probes maintain the same statistical distribution as the original variables, knockoffs maintain the same correlation structure between the original variables and the target variable.

Future work could also apply the techniques developed here to heterogeneous ensembles of feature selectors, where a different feature selector is applied to each sample of the training data. Using different feature selectors would introduce more diversity into the ensemble and so may produce enhanced results.

## Conclusions

This study has demonstrated the use and validity of data-driven thresholding methods applied to ensemble feature selectors, to provide more stable, and therefore more reproducible, selections of features than individual feature selectors, without loss of performance. Several methods of data-driven thresholding have been used in a novel context and have been shown to perform well. The use of a data-driven threshold eliminates the need to choose a threshold a-priori and can select a more meaningful set of features. A reliable and compact set of features can produce more interpretable models by identifying the factors that are important in understanding a disease [1] and can also lead to the development of more cost-effective procedures for identifying patients at risk of a disease.

A number of multivariate feature selectors were tested for use in feature selection ensembles, in contrast to the univariate filters that are typically employed in this context. Issues arising from the application of the data-driven thresholds to heterogeneous data and multivariate feature selectors, particularly those that select a subset of features rather than returning a score for each feature, were overcome, allowing these methods to be used in feature selection ensembles.

The ability to produce more stable selections of features means that clinicians can have more confidence in the results produced by machine learning models. Features that are predictive of Alzheimer's disease have been selected from the models developed here and these are in keeping with findings in the AD literature.

## Data Availability

The datasets analysed during the current study are available on reasonable request by applying to the Sydney Memory and Ageing Study via memory@unsw.edu.au and to the Alzheimer’s Disease Neuroimaging Initiative at https://adni.loni.usc.edu/data-samples/access-data/.

## Abbreviations

AD: Alzheimer’s disease
ADNI: Alzheimer’s Disease Neuroimaging Initiative
KDE: Kernel Density Estimation
MAS: Memory and Ageing Study
MA: Medrank Algorithm
MRI: Magnetic resonance imaging
PET: Positron emission tomography
RRA: Robust Rank Aggregation
TA: Threshold Algorithm
